# Quaternary ammonium silane (k21) based intracanal medicament triggers biofilm destruction

**DOI:** 10.1186/s12903-021-01470-x

**Published:** 2021-03-12

**Authors:** Esther Sook Kuan Kok, Xian Jin Lim, Soo Xiong Chew, Shu Fen Ong, Lok Yin See, Siao Hua Lim, Ling Ang Wong, Fabian Davamani, Venkateshbabu Nagendrababu, Amr Fawzy, Umer Daood

**Affiliations:** 1 Division of Clinical Dentistry, Schoolof Dentistry, International Medical University Kuala Lumpur, Kuala Lumpur, Malaysia; 2 Faculty of Biomedical Science, School of Health Sciences, International Medical University, Kuala Lumpur, Malaysia; 3 Department of Preventive and Restorative Dentistry, College of Dental Medicine, University of Sharjah, Sharjah, UAE; 4 UWA Dental School, University of Western Australia, Nedlands, Australia

**Keywords:** Calcium hydroxide, Chlorhexidine, Dentine, Intracanal medicament, Quaternary ammonium silane

## Abstract

**Background:**

Compare antimicrobial efficacy of a quarternary ammonium silane (QAS)/k21 as an intracanal medicament against *E. faecalis* and *C. albicans* biofilms formed on root dentin.

**Methodology:**

Dentin blocks were sterilized and *E. faecalis* and *C. albicans* microbial colonies were counted for colony-forming-units against 2%k21, 2%CHX and Ca(OH)2 medicaments. Biofilm colonies after 7 days on dentin were analysed using confocal laser scanning microscopy with live/dead bacterial viability staining. TEM was done to study dentin collagen matrix. Dentin discs from 3rd day and 7th day well plate was used for Raman spectra and observed under fluorescent-microscope. Docking studies were carried out on MMP-2 S^1^ binding-domain with k21**.**

**Results:**

There was reduction of *E. faecalis*/*C. albicans* when k21, chlorhexidine and calcium hydroxide were used with highest percentage in 2%k21 treated specimens. 2%k21 showed dense and regular collagen network with intact cross-banding and decreased Raman intensity for 2%k21 on 3rd day. NaOCl + k21 showed least adherence, whereas saline groups showed highest adherence of *E. faecalis* and *C. albicans* to root-canal dentin. Alizarin red staining of hDPSCs revealed calcium deposition in all groups with significant difference seen amongst 2%k21 groups. MMP-2 ligand binding was seen accurately indicating possible target sites for k21 intervention.

**Conclusion:**

2%k21 can be considered as alternative intracanal medicament.

## Background

There is acidic dissolution of dental hard tissues due to breach of microorganisms [1] through exposed dentinal tubules and cracks in teeth reaching the pulp space, leading to dental infections [2]. Poor disinfection procedures result in bacterial penetration within the root canal system leading to endodontic failures [3]. The use of voluminous irrigating solutions is considered pivotal for root canal therapy [4]. Hence the use of disinfectants becomes invaluable for the elimination of toxic microorganisms [5]. Proper disinfection helps in achieving predictable long-term outcome of root canal treatment [6]. There have been many approaches introduced to reduce the microorganism load in the root canal system, including instrumentation, root canal drug treatments (using irrigants and /or intracanal medicaments), root canal irrigation systems (e.g. ultrasonically activated irrigation, EndoVac) and root canal sealers [7–9]. Intracanal medicaments have contributed mainly towards root canal disinfection by promoting significant microbial reduction after chemo-mechanical procedures [10–13]. Hence the intracanal medicament plays a key role in root canal treatment. Calcium hydroxide and chlorhexidine (CHX) are commonly used intracanal medicaments for root canal disinfection.

*Enterococcus faecalis* is highly resistant and has a pivotal role in infections of root treated teeth leading to high rate of treatment failure [14]. *E. faecalis* is the most common microorganism present in persistent intraradicular infections compared with primary chronic periapical periodontitis [15]. *Enterococcus faecalis* has the ability to survive in high alkalinity and in presence of antimicrobial agents [16]. The antimicrobial agents used can only reduce *E. faecalis* but cannot remove all microbes colonized inside the root canal effectively [17]. Therefore, it is imperative to analyze different formulations with minimal toxic effects on periapical tissues and substantial antimicrobial potential. *Candida* occurs in small proportion of root canal infections with prevalence of 8.20%, possessing virulence factors that may play a role in the onset of endodontic pathologies [18, 19]. Therefore, it is critical to find new methods and antimicrobial agents for effective control of bacterial and fungal infections in root canal therapy.

Several investigations have been conducted to determine the antimicrobial properties of dental materials. Polymeric and inorganic agents are capable of inhibiting microorganism proliferation [20]. Quaternary ammonium-based materials are used as antimicrobial agents showing its potential as new bioactive monomers. These materials exhibit excellent growth inhibitory effect against most microorganisms which is comparable to most antibiotics used [21]. In this study, a new class of Organosilane quaternary ammoniums (k21(QAS)); KHG *FiteBac® Technology, GA, USA*) was investigated containing one molecule of 3-(trimethoxysilyl) propyldimethyloctadecyl ammonium chloride (SiQAC) and three molecules of 3 methacryloxypropyltrimethoxysilane which were attached with a silane-based sol–gel route having an anchoring unit consisting of tetraethoxysilane [22]. The compound is a contact killing agent (*kill*-on-*contact* microbiocidal activities) possessing low cytotoxicity and broad-spectrum antimicrobial activities [23–26]. The antimicrobial property is derived from –C_18_H_37_ lipophilic alkyl chain which disturbs the bacterial membrane causing total cell autolysis. The binding of the quaternary ammonium compound is selective and towards the Sortase A protein within the bacterial membrane via electrostatic and hydrogen bonding coupled with van der Waals’ interactions. The compound interferes with the catalytic activity of Sortase A, resulting in reduced anchoring of cell surface proteins [27, 28]. Data suggests, quaternary ammonium compounds are active even at low concentrations [29]. The compound is partially condensed and condenses fully on application to dentine in presence of dentine fluids and moisture. The presence of silane from tetraethoxysilane utilises tetraethoxysilane (TEOS) or dimethyldiethoxysilane as the anchoring unit and improves attachment, a fact that may be as superior to Salvizol, a previous QAC based root canal disinfection [30]. The compound is dissolved in ethanol or acetone to produce a cavity disinfectant for intraoral use. Apart from its antibacterial ability, k21 also demonstrates anti-MMP activity [31] and optimum bond strength after its application to etched dentine prior to adhesive bonding [23]. Anti-MMP properties inactivate dentin proteases increasing the biomechanical resistance of collagen resulting increased collagen matrices [32]. Importantly, MMP-2 was mainly identified in demineralized dentin suggesting its potential role in dentin extracellular matrix degradation during the carious process and breakdown of dentin collagen matrix, that can result in leaving native triple-helical type I collagen into ¾ and ¼ fragments at the Gly-Leu/Isoleu peptide bond and telopeptides [31–33]. The compound leaves behind an immobilized 3D amphiphilic network in the dentinal tubules for its long-term antibacterial property [33]. Daood et al*.* [25] concluded that 2% k21 does not have cytotoxic effects on 3T3 NIH mouse fibroblast cells and has antimicrobial efficacy when used in conjunction with sodium hypochlorite (NaOCl) as root canal irrigants against *E. faecalis* [26]. To the author’s knowledge, so far, no study has been conducted to investigate the potential of k21 as an intracanal medicament.

Identification of bacteria in different conditions have been conserved based on morphological evaluation. Vibrational Raman spectroscopy have been pivotal in displaying fingerprint regions for identification of chemical composition of bacterial biofilms [24, 26]. Therefore, this pragmatic approach was applied to identify changes within the bacterial biofilms based on changes in the biofilm matrix and bacterial membrane destruction. The aim of the current study (preliminary) was i) to compare the antimicrobial efficacy of k21 as an intracanal medicament against *E. faecalis* and *C. albicans* biofilms formed on root dentine, ii) to evaluate biofilm changes with k21 against *E. faecalis* in an in vitro tooth model using Raman spectroscopy analysis and MMP-2 analysis via molecular docking. The null hypotheses tested were: (i) k21 has antimicrobial efficacy on *E. faecalis* and *C. albicans* biofilms compared to commonly used intracanal medicaments, (ii) k21 influences composition of biofilm backbone compared to chlorhexidine and calcium hydroxide against *E. faecalis* biofilm.

## Methods

The k21 intracanal experimental formulation was prepared via a sol–gel route (KHG *FiteBac® Technology*). The study protocol was approved by Institutional Research Ethical Committee (IMU-JC NO BDS I-01-2019(04), BDS 1-01-2019(11)].

### Antimicrobial assessment: colony forming units

#### Preparation of dentine blocks

Eighty freshly extracted human single rooted teeth were used for this experiment. Rotary diamond discs in a straight handpiece were used to decoronate teeth below cemento-enamel junction and apical part of the root to obtain 6 mm of middle third of root. Gates Glidden drill No.3 was used to standardize the internal diameter of the root canals. To remove debris, teeth samples were placed in an ultrasonic bath containing 17% ethylenediaminetetraacetic acid (EDTA) for 5 min followed by 3% sodium hypochlorite (NaOCl). The dentine blocks were cleaned by using Digital Ultrasonic Cleaner Machine (Fisher Scientific). Dentine blocks were then sterilized by using autoclave machine (TOMY Digital Biology) [34, 35]. In other samples, SEM was used to confirm no morphological changes on dentin and dentinal tubules induced by sterilization (data not shown). Also, while performing the experiments, a small extra sample was inoculated to a culture plate, other than the inoculated bacteria was identified (for all experiments).

#### Contamination of dentine blocks

Dentine blocks were contaminated with *E. faecalis* (n = 40) and *C. albicans* (n = 40). ATCC 29219 *E. faecalis* and *C. albicans* were cultured in Trypticase Soy Medium (Santamaria, CA, USA) and Sabouraud Dextrose Agar (SDA) medium (Oxoid) respectively. *E. faecalis* colony was grown at 37 °C in respective medium to obtain microbial suspension. About 10 mL of microbial suspension (0.5 McFarland standard) was taken in a sterile tube for *E. faecalis* and *C. albicans*. Sterilized dentine blocks were placed into the tubes in an aseptic environment, along with the microbial suspension and allowed to grow for 2 days in a BSL II laminar flow hood (Bioair Safemate, Italy). Fresh microbial broth was replaced every third day for 21 days. The sterility control was maintained by using the broth without the microorganisms as matched control, and a clear solution was observed till the end of the experiments. Alternatively, for the experimental group a portion of broth with the microbes from the experimental set up was streaked on the plated.

#### Antimicrobial assessment

The blocks were randomly assigned into four groups (n = 10); Group I: 2% k21, Group II: 2% chlorhexidine digluconate (CanalPro, COLTENE), Group III: 41% calcium hydroxide (CALASEPT Plus, DIRECTA), Group IV: saline (negative control). After 21 days of contamination, the medicaments were placed inside the canals and sealed at both the ends with paraffin wax and incubated in an anaerobic environment set up in a sealed candle jar incubated at 37 °C for 5 days. After 5 days, dentine debris was removed from the canals by using Gates Glidden drill No.4 and No.5, which corresponds to 200 µm and 400 µm depth of dentinal tubules respectively. Dentine shavings were collected in 1 mL of half strength BHI broth for *E. faecalis* and Sabouraud Dextrose broth for *C. albicans*. The shavings were vortexed, and the supernatant was serially diluted and plated on BHI agar or SD Agar for *E. faecalis* and *C. albicans* respectively. The plates were incubated in 5% carbon dioxide incubator at 37 °C for up to 24 h. Colonies were counted and readings were tabulated as colony forming units (CFU).

### Confocal Laser Scanning Microscopy Assessment (CLSM)

#### Preparation of dentine blocks

The roots of teeth were removed using a low-speed, water-cooled, diamond saw (Buehler, Lake Bluff, IL, USA) and cut to expose deep dentine to prepare 1.5 mm dentine discs. A total of 80 dentine discs, 40 for single colony groups (*E. faecalis and C. albicans*) and 40 for dual colony were prepared. Dentine discs were wet-polished (600–120) using SiC papers (Carbimet, Buehler, Lake Bluff, IL, USA), rinsed with deionized-water for 5 min and polished by gentle blotting using absorbent paper (Kimwipes; Kimberly-Clark Professional, Roswell, GA, USA). To remove debris, teeth samples were placed in an ultrasonic bath containing 17% ethylenediaminetetraacetic acid (EDTA) for 5 min followed by 3% sodium hypochlorite (NaOCl); dentine blocks were later autoclaved at 121 °C for 15 min and the blocks were vertically anchored in 24-well culture plate using metal devices.

#### Biofilm formation on dentine

*E. faecalis* (#29212, ATCC Manassas, VI) was inoculated in Tryptone Soya Medium broth and incubated at 37 °C for overnight. The cells were grown on the surface of prepared BHI plates and bacterial cells were re-suspended in BHI broth and adjusted to a turbidity equivalent to a 0.5 McFarland Standard, this broth was used as a standard bacterial inoculum. The dentine blocks were placed individually in sterile 24-well tissue culture containing the bacterial suspension plates (Thermo Fisher Scientific Inc.), and incubated (37 °C with 5% CO_2_) for 3 days to enable bacterial adhesion. *C. albicans* cells (− 80 °C stock) were propagated in Sabouraud dextrose agar plates and incubated overnight at 30 °C in an orbital shaker at 180 r.p.m. and grown for 14–16 h at 37 °C to a turbidity equivalent of 0.5 McFarland Standard. The standard *C. albicans* culture was mixed and transferred to the dentine blocks individually in sterile 24-well tissue culture plates and grown for 3 days at 37 °C with 5% CO2. Dual colonies of both species were incubated at a ratio of 50/50 with Trypticase soy and Sabouraud dextrose broths.

The dentine discs were washed with sterile saline solution to remove any unattached microbial cells. Group I contained dentine discs treated with saline, served as a control group. In groups II, III, and IV, the prepared dentine specimens underwent treatment with 2% chlorhexidine, 41% Calcium hydroxide, and k21 respectively using a sterile saturated micro brush (Dentsply/DeTrey; Konstanz, Germany) and rubbed for 20 s. After treatment, dentine discs were rinsed gently with saline to remove the medicaments. The specimens were returned immediately to the well plates containing bacterial suspension inside an anaerobic sterile incubator at 37 °C. The microorganisms were allowed to redevelop for another 4 days. All application procedures were carried out in a sterile environment.

#### Confocal microscopy assessment

Confocal laser scanning microscopy (CLSM; Fluoview FV 1000, Olympus, Tokyo, Japan) was performed to examine bacterial biofilm after secondary adhesion phase (7 days). The excitation wavelength was adjusted to 488 nm with 10 × objective lens. Baclight bacterial viability (molecular probe #L7012 LIVE/DEAD BacLight stain Invitrogen) was used to stain live/dead bacteria (between 500 and 550 nm) [36]. Bacterial images within the microcolony were taken randomly with Z-stack rendered over a 25- μm-thickness. Five images stacks per biofilm concentration inside one group were taken as data sets for calculation of results for both single and dual species on a two-dimensional x–y section based on color segmentation algorithms and examined using bioimageL software (v.2.0. Malmő, Sweden).

### Transmission Electron Microscopy of Dentine Collagen

After secondary adhesion phase, transmission electron microscopy (TEM) characterization was done on dentine discs to study the structural variations of dentine collagen matrix. Dentine discs (*n* = *5*) were rinsed with deionized water for 5 min and fixed after buffering with 0.1 M sodium cacodylate for 1 h. The specimens were treated with 1% osmium tetraoxide and later dehydrated using ascending concentrations of ethanol. The specimens were than infiltrated with araldite resin and later subjected to ultra-microtome to cut ultrathin sections (90~ nm) with a diamond knife. The sections were collected on the grids and stained with uranyl-acetate, UO2(CH7COO)2·2H2O for 10 min and analysed using TEM at 100 kV (JEOL-1010, Japan) [36].

### Changes in composition of E. faecalis biofilm

#### Dentine blocks preparation and contamination with E. faecalis

Forty-eight non-carious mandibular premolar were collected and stored in 0.5% chloramine T solution. Teeth were decoronated and sectioned vertically to obtain 3 × 3 × 1.5 mm dentine discs from the middle third of the root using a diamond-impregnated disc with water coolant (Buehler Iso Met low speed cutting machine, Lake Bluff, IL, USA). The samples were immersed into ultrasonic bath (Labsonic P, Braun Biotech International, Goettingen, Germany) at 30 kHz for 15 min to remove debris which allowed *E. faecalis* (n = 24) to penetrate. Dentine discs were then autoclaved (131 °C, 20 min) and stored in saline before the inoculation with *E. faecalis*.

#### Raman analysis

On the 3rd day after inoculation, microbial suspension was discarded after the incubation period. Sterile saline was used to gently irrigate dentine disc for one minute to remove the loosely bound *E. faecalis*. Following this, dentine discs were placed into a new 50 ml test tube with saline for sonication at 37 kHz (5 min). This procedure effectively dislodged loosely bound *E. faecalis* and the biofilm. Twenty-four dentine discs were randomly assigned into 4 groups. Group I dentine discs (n = 6) were treated with saline and not treated with any intracanal medicament. Groups II, III and IV, (n = 6/group) were treated with 2% k21, calcium hydroxide and chlorhexidine respectively by immersing dentine discs into medicaments in a 24-well culture plate for 30 min. After treatment, dentine discs were rinsed gently with saline to remove the medicaments. Three random dentine discs from each group were placed in a new 24-well culture plate and fixed with 2.5% glutaraldehyde (2 ml) for 2 h at 4 °C. The specimens were then sent for Raman analysis. Remaining dentine discs from each group were then returned to separate 50 ml test tubes containing fresh TSB medium and placed inside anaerobic orbital shaker incubator for 4 more days to allow for redevelopment. TSB medium was changed every 3 days. On the 7th day, dentine discs from each group were washed gently for one minute with saline, placed into separate 50 ml test tube with saline and sonicated at 37 kHz for 5 min. Following this, dentine discs were placed inside 24-well plates, fixed with 2 ml of 2.5% glutaraldehyde for 2 h at 4 °C and sent for second Raman analysis.

#### Raman data acquisition and processing

For Raman measurements, well plated dentine discs from 3rd day and 7th day were dried for 15 min at 35 °C and transferred to low fluorescent microscopic slides. The spectra were recorded randomly across all dried biofilm specimens in an area of 5 µm at 785 nm wavelength using Raman spectrometer (Nanocat, University of Malaya) equipped with a Leica microscope and lenses (JY LabRam HR 800, Horiba Jobin Yvon, France) with curve-fitting Raman spectroscopy software (Labspec 5) [26]. An argon ion 632.8 nm laser at a < 500 µW power and a 100 × objective (a superior signal/noise ratio) were utilized for an exposure of 60 s in an X and Y directions within 200 cm^−1^ and 3200 cm^−1^. All information rich regions were recorded at room temperature and in dark to avoid background noise (background peaks) due to ambient light. The Raman peaks for ECM rich biofilm matrices were centered at 484 cm^−1^ and 584 cm^−1^ and monitored for stabilization.

### Adherence assay

#### Preparation of dentine blocks and treated with medicaments

Dentine blocks (n = 80) measuring 3 × 3 mm were obtained from single rooted human mandibular premolars. Dentine blocks were cut using a slow-speed isomet (Buehler IsoMet Low Speed, Lake Bluff, IL, USA). Dentine blocks were then washed and cleaned with deionized water for 30 min and autoclaved. Eighty samples were randomly divided into 8 groups (n = 10): group 1, 6% NaOCl with 2% k21 (DM Healthcare Products Inc., San Diego, Ca, USA); group 2, 6% NaOCl with 41% Calcium hydroxide (Directa AB, Upplands Vasby, Sweden); group 3, 6% NaOCl with 2% chlorhexidine (Coltene, Altstaten, Switzerland); group 4, 6% NaOCl with saline; group 5, 2% k21; group 6, 41% Calcium hydroxide; group 7, 2% chlorhexidine; group 8, saline. The blocks were immersed in tubes containing intracanal medicaments. Saline was used between NaOCl and different medicaments. The volume and time of all the intracanal medicaments was standardized to 2 mL and 30 min respectively.

#### Contamination of dentine blocks

Further each group was randomly divided into two subgroups (*E. faecalis and C. albicans*). Dentine blocks were contaminated with *E. faecalis* (n = 40) and *C. albicans* (n = 40). All dentine blocks were inoculated with 2 mL microbial (*E. faecalis* and *C. albicans*) suspension in a 24 well plate and incubated at 37 °C for 72 h in an orbitary rotary incubator (Yihder Technology Co. Ltd, Xinbei City, Taiwan). The microbial suspension was discarded, and loosely bound microorganisms were removed from dentin blocks using sterile PBS. Later, dentin blocks were sonicated to dislodge the biofilm in PBS at 37 kHz for 5 min.

#### Adherence assessment

Dentin blocks were stained and counterstained with acridine orange (AO) (Sigma-Aldrich, St. Louis, MO, USA) and 0.1% crystal violet (Sigma, St. Louis, MO, USA) for 15 and 5 min respectively. The blocks were observed under fluorescent microscope (Eclipse TiU, Nikon, Tokyo, Japan) [37, 38]. An independent blinded observer examined five different dentin blocks at three randomly selected areas. The Image J software (National Institutes of Health, Bethesda, MD, USA) was used for bacterial counts [38].

### Alizarin Red Staining

Human dental pulp stem cells (DPSCs) were isolated following the protocol followed by Daood et al*.* (23). The cells were allowed to grow inside Dulbecco’s modified Eagle’s medium (DMEM) which was supplemented using 1% non-essential amino acids, 20% fetal bovine serum (FBS) (Gibco-Invitrogen, USA) and 1% penicillin–streptomycin (Pen-Strept). Dentine discs (*n* = *3*) of 1.5 mm each were prepared from freshly extracted teeth. After applying the medicaments on the occlusal surface of the discs, the specimens were washed and placed in cell medium with cell growing on the pulpal side for 3 days. Dentine discs were washed with PBS twice and fixed with 70% ethanol for one hour. After washing the specimens, Alizarin red (Sigma Aldrich; Suite#3, Brisbane-Australia) was used to stain the cells (20 min with 40 mmol/l) adjusted with a pH of 4.5 with ammonium hydroxide. Light microscopy (LW 0.52 Nikon Japan, Eclipsee) was used to take representative photographs. The cells were mixed with alizarin red solution using the tip of a Pasteur pipette and observed under light microscopy within 3 min for qualitative examination. Positive alizarin red staining was graded according to the extent of heavy red stained clumps observed per high power field under ordinary light microscopy under original magnification of 10 × . The nodules from the cells on pulpal side of the discs were solubilized for 20 min and incubated with 10% cetylpyridinium chloride (Sigma–Aldrich; Suite#3, Brisbane-Australia). The mean value of control groups (100% staining) was used to calculate the absorbance of the resulting solution after 3-days.

### Molecular simulation for understanding of MMP action

The present study was undertaken to determine whether k-21 binds to MMP-2 enzyme via computational molecular docking simulations. Docking studies were carried out on a crystal structure, MMP-2 S^1^ binding-domain complexed (chain B, PDB ID: 6LZG). Glide (Standard precision (SP) mode and Extra precision (XP) mode) and Induced-Fit docking modules of Schrodinger 2019-4 were used to evaluate the interactions between k21 and MMP-2 (MMP-2 (PDB ID: 3AYU) binding domain. For docking analysis of proteins, ‘Protein preparation’ module of Schrödinger with default settings was operated with water molecules with less than three hydrogen bonds were removed. Energy was minimized using OPLS_2005 force field adding hydrogen bonds (pH 7.0) with missing side chain atoms and loops within the protein structure. MMP-2 was arranged without inbound ligand.

### Statistical analysis

The Wilcoxon signed-ranks test was used to check the difference in CFUs between the 200 and 400 μm depths (*P* > 0.05). Data for adherence assay was analysed (GraphPad Prism 8 XML Project) using Ordinary one-way ANOVA test followed by Dunnett’s multiple comparisons test. Data for Raman shift (cm^−1^) was presented as mean ±  standard deviation and analysed using a statistical package (IBM SPSS statistics software, version 23.0 IBM Corp. Armonk, NY, USA). One-way ANOVA followed by Post-hoc Tukey was conducted to determine significant differences amongst the four groups of intracanal medicaments and control (a = 0.05). All statistical analysis used a 95% confidence limit, so that *p* values ≥ 0.05 are not considered statistically significant. The values were tested for normality using Shapiro–Wilk test and homoscedastic (modified Levene test).

## Results

Table 1 showed number of colony forming units for *E. faecalis* and *C. albicans* collected from the dentine chips at 200 µm and 400 µm depth of dentinal tubules for k21, chlorhexidine, calcium hydroxide and the saline (control) groups. There was no bacterial growth at 200 µm and 400 µm depth, when treated with 2%k21, chlorhexidine and calcium hydroxide showing 100% reduction when used as an intracanal medicament for 5 days. *E. faecalis* and *C. albicans* were present at both the depths when treated with saline.

**Table 1:** Mean and Standard Deviation (SD) of the Colony forming units counted and readings tabulated after treating with various medicaments at 200 and 400 µm level. The results indicate that there was a 100% reduction of *E. faecalis* and *C. albicans* when k21, chlorhexidine (CHX) and calcium hydroxide were used as intracanal medicament for 5 days

Groups	*Enterococcus faecalis*		*Candida albicans*	
	Mean ± SD		Mean ± SD	
	200 µm	400 µm	200 µm	400 µm
2%k21	0	0	0	0
Chlorhexidine (CHX)	0	0	0	0
Calcium hydroxide	0	0	0	0
Saline	4.0 ± 1.41^a^	12.0 ± 3.43^a^	2.5 ± 1.269^a^	3.6 ± 2.17^a^

**Fig. 1:**
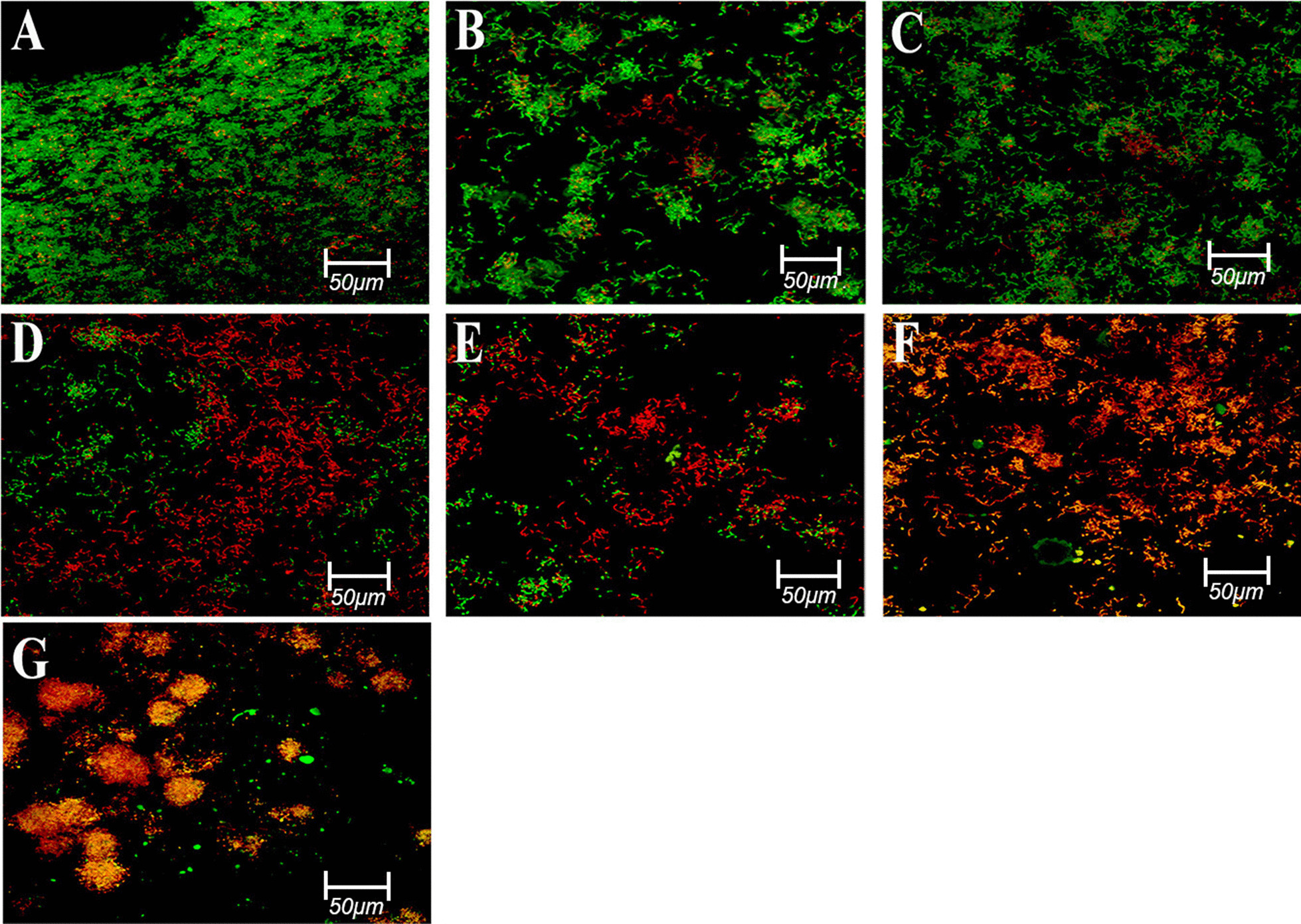
Representative confocal images of *E. faecalis* biofilms of different groups stained using live/dead bac light bacterial viability kit; (**A**, **B**) control after 3 days; (**C**) control after 7 days; (**D**) 2%CHX after 7 days; (**E**) 41% Ca(OH)2 after 7 days; (**F**) 2%k21 after 3 days; and (**G**) 2%k21 after 7 days. Excitation was performed at λ = 514 nm. Green indicates a high level of bacterial viability in control specimens. Most of the red fluorescence in 2%k21 specimens indicating dead cell population. Scale bar = 50 μm

CLSM images representing *E. faecalis* biofilm experimental groups following treatment with different intracanal medicaments were shown in Fig. 1. The control untreated specimens showed densely clustered green colonies with minimal areas of dead bacterial cells (*p* < *0.05*) after 3 (Fig. 1A, B) and 7 days (Fig. 1C) of growth. The 2%CHX (Fig. 1D) and 41% Ca(OH)_2_ (Fig. 1E) specimens after 7 days showed carriable areas of dead cell population. Predominantly, the bacteria in 2%k21 treated specimens showed red/orange fluorescence indicating mostly dead cells (*p* < *0.05*) after 3 (Fig. 1F) and 7 days of growth (Fig. 1G) (percentage data not shown). Biofilms decreased significantly with a change (*p* < 0.05) in intracanal medicaments from 2% chlorhexidine to 2%k21. The cell number was notably increased in control and 2% chlorhexidine specimens in contrast to the 2%k21 specimens after three days (*p* < 0.05), that was coincident with the fact that there is incapability to form biofilm (Table 2; percentage ratios data not shown).

**Table 2:** Data showing different specimens using 2%k21, chlorhexidine (CHX) and Calcium hydroxide (Ca(OH)2) using Fisher’s protected least significant difference showing the cell number was notably increased in control and 2% chlorhexidine specimens in contrast to the 2%k21 specimens, a fact that is coincident that there is incapability to form biofilm as clear with the *p* values. The significant *p* values are noted as bold with log difference calculated for bacterial counts. The symbols (ɸ) used indicated significant differences and similarities amongst different groups other than the bold ones

Biofilm Specimens	1	2	3	4	5	6	7	8	9	10
*n* = *5*										
*Enterococcus faecalis*-*Albicans*/control	*p* = 0.080 ɸ									
	*p* = 0.076 ɸ	p = 0.002 6.16 × 10^9^								
	***p*** **= 0.031** 5.11 × 10^11^	***p*** **= 0.001**	*p* = 0.154							
*n* = *5*										
*Enterococcus faecalis-Albicans*/2%CHX	***p*** ** = 0.05**	*p* = 0.3	*p* = 0.3	*p* = 0.2						
	*p* = 0.7 ɸ	*p* = 0.54 ∞	*p* = 0.12	*p* = 0.04	*p* = 0.05					
	*p* = 0.02	*p* = 0.003	*p* = 0.2	*p* = 0.3	*p* = 0.4	***p*** **= 0.01**				
*n* = *5*										
*Enterococcus faecalis-Albicans*/ Ca(OH)2	***p*** **= 0.01**	*p* = 0.05	*p* = 0.05	*p* = 0.7 ∞	*p* = 0.6 ∞	*p* = 0.5 ∞	***p*** **= 0.04**			
	*p* = 0.7 ɸ	*p* = 0.22	*p* = 0.04	*p* = 0.55 ∞	*p* = 0.8 ɸ	*p* = 0.3	*p* = 0.4	*p* = 0.2		
	*p* = 0.05	*p* = 0.001	*p* = 0.7 ɸ	*p* = 0.9 ɸ	*p* = 0.01	*p* = 0.3	*p* = 0.2	*p* = 0.1	*p* = 0.1	
*n* = *5*										
*Enterococcus faecalis-Albicans*/2%k21	***p*** **= 0.05**	***p*** **= 0.05**	***p*** **= 0.05**	*p* = 0.55	*p* = 0.08	*p* = 0.1	***p*** **= 0.01**	***p*** **= 0.05**		
	***p*** ** = 0.02**	*p* = 0.11	*p* = 0.3	*p* = 0.04	*p* = 0.34	***p*** **= 0.02**	***p*** **= 0.01**			
	*p* = 0.02	***p*** **= 0.03**	***p*** **= 0.005**	*p* = 0.06	***p*** **= 0.03**	***p*** **= 0.04**				

**Fig. 2:**
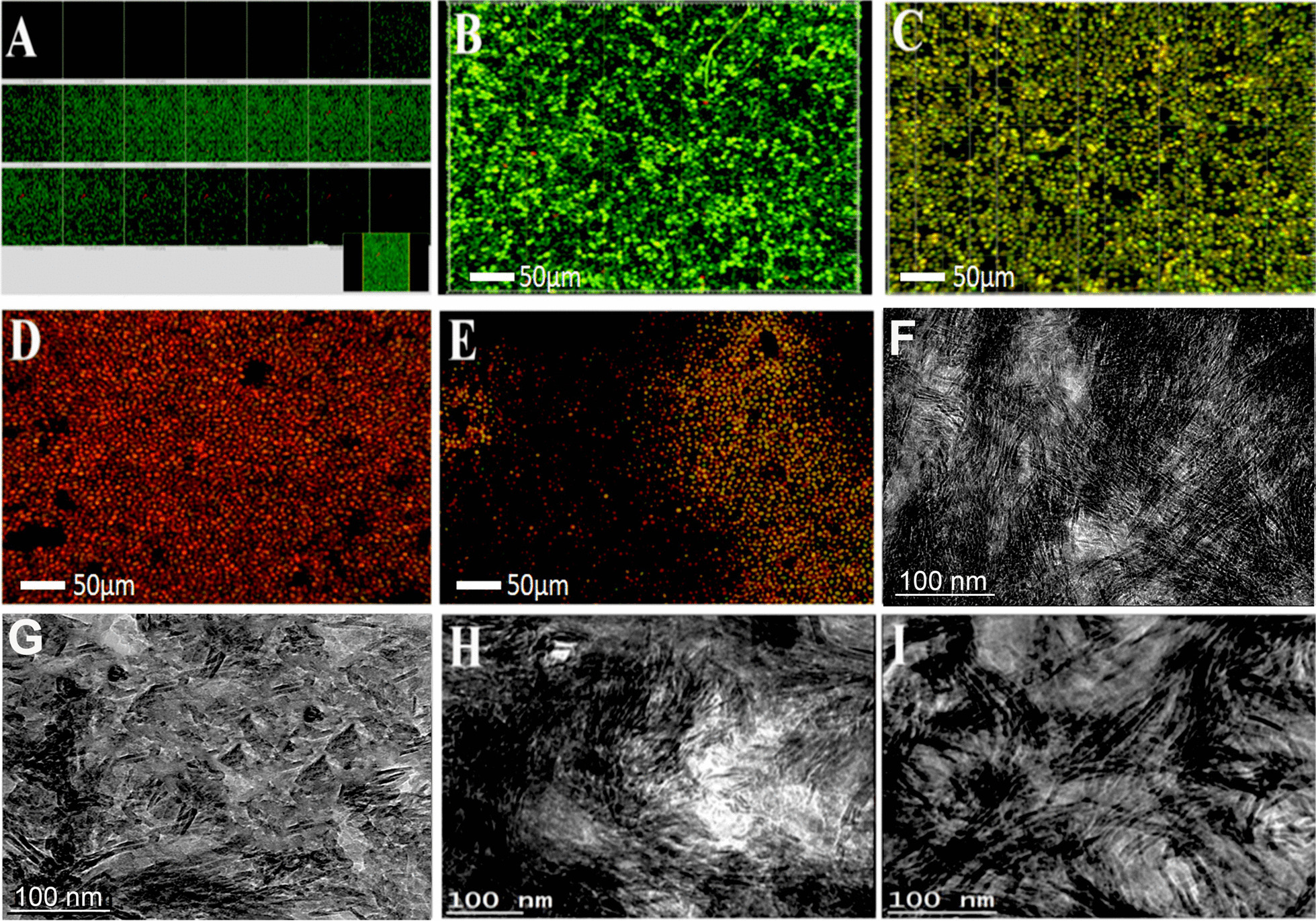
Representative confocal images of *Candida albicans* (**A**, **B**) control group cells (in green) and (**C**) 2%CHX treated biofilms. (**D**) Specimens treated with 41% Ca(OH)2; (**E**) Structural destruction of *Candida albicans* micro-colonies after treatment with 2%k21 disinfectant. (**F**) degraded collagen fibers seen in after enzymatic degradation showing presence of denatured collagen fibers; TEM showed non-intact collagen fibres suggesting a significant degradation effect of bacterial biofilm in the control group. The collagen fibres were thinner and irregular in contour as compared to the medicament-treated specimens. (**G**) 2%CHX; (H) 41% Ca(OH)2; (**I**) 2%k21 where an increased number of fibrils seen in a relatively uniform manner. (**G**): Specimen treated by 2% chlorhexidine and (**H**) specimen treated by 41% calcium hydroxide both showing improved collagen fibrils compared to control. However, both 2%CHX (**G**) and calcium hydroxide (**F**) showed isolated fibrils and irregular arrangement of substructure. (**I**) Specimens treated by 2% k21 showing increased number of collagen fibrils and well demarcated collagen cross banding pattern. This could be due to the ability of 2%k21 as an inhibitor to MMP and cysteines cathepsins leading to increased resistance of dentine collagen against degradation and ingress by bacteria leading to better preservation of the collagen fibrils framework

Representative CLSM images of *C. albicans* within the root dentin in different groups has been presented in Fig. 2. Representative confocal images of *Candida albicans* (Fig. 2A, B) control group cells (in green) showed predominant growth of candida biofilms. The predominant areas of dead *C. albicans* was found in 2%k21 specimens (Fig. 2E). This was higher than the chlorhexidine (Fig. 2C) and lesser than calcium hydroxide (Fig. 2D) groups. There was structural destruction of *C. albicans* microcolonies after treatment with 2%k21 disinfectant.

TEM micrographs in Fig. 2F showed non-intact collagen fibers indicating a significant degradation effect of the bacterial biofilm. The collagen fibers appeared thinner and in irregular contour as compared to 2%k21 treated specimens where increased number of fibrils were seen in a relatively uniform manner. Specimen treated by 2% chlorhexidine displayed isolated and irregular collagen fibrils compared to control. Collagen network in 2%k21 specimens were seen denser and regularly arranged with intact cross-banding forming more organized network structures indicating reduced effect of biofilm onto the dentin specimens (Fig. 2I). The TEM investigation confirmed deep delivery and maybe a close association/attachment of k21 was seen. Collagen fibrils were also seen preserved in 41% calcium hydroxide (Fig. 2H) treated specimens.

**Fig. 3:**
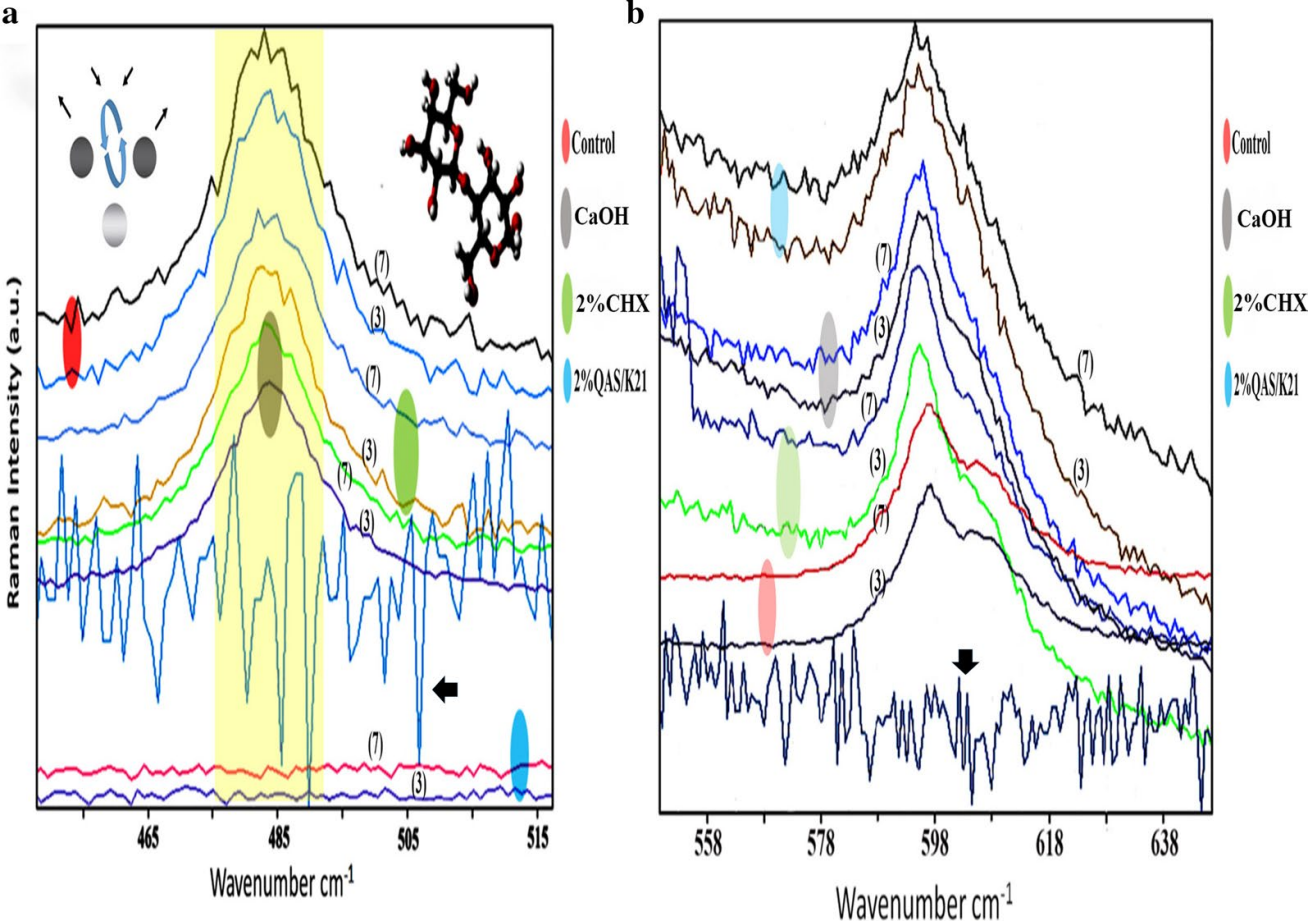
Raman spectra of *E. faecalis* biofilms on dentin disc specimens treated with different intracanal medicaments protocol. (**a**) Significant spectral differences of control and treated specimens can be seen in the 480–490 cm^−1^ region after normalization (region highlighted yellow). The Raman intensity on the y-axis represented the amount of amylopectin exopolysaccharide detected in an arbitrary unit, (3) represented the Raman analysis from dentin disc samples sent on the 3rd day while (7) represented samples from 7th day. In the 2%k21 case, the Raman fit was applied to the spectrum. While calculating, the weak curved sections, it was subtracted from the main spectrum producing the flattest baseline for 2%k21 at the same carbohydrate fingerprint region. The two spectra fall on the red, green, grey and blue regions belonging to the Raman intensity created by dentin disc specimens in control group, 2%CHX, Ca(OH)2 and 2%k21 respectively in A; and red, green, grey, and blue control group, 2%CHX, Ca(OH)2 and 2%k21 respectively in B. Raman peak acquired in same region during calibration process with silicon wafer indicated with black arrow showing remarkable difference between testing peaks and experimental groups. (**b**) Raman shift from 598–588 cm^−1^ presented the Raman effect caused by β-galactosidase. The β-COC ring deformations are the mode of vibrations due to the strong coupling due to the glycosidic ring skeletal deformations of the carbohydrate. The phospho-β-galactosidase is found in bacterial cytoplasm

All Raman shifts were recorded and converted into mean value with standard deviation as shown in Fig. 3. Raman intensities in the four experimental groups had showed obvious and inter-related variations at regions around 484 cm^−1^ and 598 cm^−1^ (Table 3), with changes verified in comparison to the Raman peak acquired in same region during calibration process with silicon wafer. In Fig. 3a, the y-axis at 480 cm^−1^–490 cm^−1^ region represented the amount of glycosidic bond in amylopectin exopolysaccharide present in the biofilm. On the third day, Raman intensities by glycosidic bond in chlorhexidine group, calcium hydroxide group, and 2%k21 group are lower than the control group suggesting all medicaments have the effect of removing exopolysaccharide hence disrupting *E. faecalis* biofilm. Raman intensity by 2%k21 on the 3rd day was the lowest and near to 0 arbitrary unit on y-axis, suggesting a near complete removal of exopolysaccharide hence complete disruption of *E. faecalis* biofilm. This was the growing phase in the *E. faecalis* biofilm as compared to Raman intensity created by calcium hydroxide on 3rd and 7th day, which was lower than chlorhexidine (Fig. 3a). This was suggestive of the fact that calcium hydroxide had more breakdown of exopolysaccharide biofilm as compared to chlorhexidine.

**Table 3:** Mean values of Raman shift where obvious Raman intensities are recorded. Raman shifts found in the region from 490 cm^−1^ until 476 cm^−1^ were all assigned to amylopectin, which is an exopolysaccharide found in biofilm. Raman shift from 598–588 cm^−1^ presented the Raman effect caused by β-galactosidase. Values: means ±  standard deviation. Groups identified by different alphabets were significantly different at *p* < 0.05. The β-COC ring deformations are the mode of vibrations due to the strong coupling due to the glycosidic ring skeletal deformations of the carbohydrate. The phospho-β-galactosidase is found in bacterial cytoplasm

Groups	Days	Raman Shift cm^−1^	Raman Shift cm^−1^
		(480–490 cm^−1^)	(588 cm^−1^ –598 cm^−1^)
2% k21	3rd	480 cm^−1^ ± 2.1 B	588 cm^−1^ ± 0.9 B
	7th	476 cm^−1^ ± 1.2 C	589 cm^−1^ ± 1.5 B
Chlorhexidine (CHX)	3rd	483 cm^−1^ ± 2.2 A	595 cm^−1^ ± 3.4 A
	7th	484 cm^−1^ ± 3.1 A	597 cm^−1^ ± 1.1 A
Calcium Hydroxide Ca(OH)2	3rd	481 cm^−1^ ± 2 B	590 cm^−1^ ± 1.9 B
	7th	480 cm^−1^ ± 3.3 B	592 cm^−1^ ± 2.9 B
Control	3rd	486 cm^−1^ ± 1.1 A	597 cm^−1^ ± 4.1 A
	7th	490 cm^−1^ ± 1.9 A	598 cm^−1^ ± 2.2 A
		β-COC ring deformations -amylopectin	β-galactosidase

The Raman intensity by glycosidic bond increased from 3rd to 7th day inside the control groups (untreated), suggesting more exopolysaccharide were being produced due to the continuous growing *phase of E. faecalis in biofilm* during the re-incubation period. Therefore, the amount of new bacteria formed and adhered to the biofilm during re-incubation period, are represented by the difference in Raman intensity by glycosidic bond at 3rd day and 7th day. The number of new bacteria formed in in calcium hydroxide was lesser than in chlorhexidine, suggesting that calcium hydroxide has a better inhibition on the growth of *E. faecalis* in biofilm.

Another obvious variation in spectra within the biofilm was the Raman shift around 588 cm^−1^ to 598 cm^−1^, which was assigned to the β-galactosidase enzyme Fig. 3b. This enzyme is found in the inner membrane of an intact *E. faecalis* for the metabolism of carbohydrate. When the membrane of *E. faecalis* is destroyed by medicament, beta-galactosidase enzyme is released into the biofilm and detected by Raman analysis. Therefore, higher Raman intensity of beta-galactosidase enzyme in biofilm, indicates more destruction of *E. faecalis*. Raman intensity by beta-galactosidase on 3rd day was highest for 2%k21 followed by > calcium hydroxide, > chlorhexidine and > control group (untreated) at 588 cm^−1^, 590 cm^−1^, 595 cm^−1^ and 597 cm^−1^ respectively, suggesting that 2% k21 has destroyed the *E. faecalis* the most, followed by calcium hydroxide and chlorhexidine.

Mean adherence values of *E. faecalis* and *C. albicans* to dentine treated with various intracanal medicaments is shown in Table 4 (representative fluorescence microscope images not shown). Group 2 (NaOCl + k21) showed least adherence, whereas Group 5 (saline) showed highest adherence of *E. faecalis* and *C. albicans* to root canal dentine. When comparing the groups with NaOCl irrigation, Group 2 (NaOCl + k21) showed significantly lesser adherence values (*p* < 0.05) compared to Group 1 (NaOCl + Saline), Group 3 (NaOCl + Calcium hydroxide) and Group 4 (NaOCl + Chlorhexidine). Besides that, when comparing group 2 (NaOCl + k21) to group 6 (k21), there was significant reduction in the adherence of *E. faecalis*.

**Table 4:** Mean and standard deviation of adherence of *E. faecalis* and *C. albicans* to root dentine treated with various intracanal medicaments

Groups	*Enterococcus faecalis*	*Candida albicans*
	Mean ± SD	Mean ± SD
NaOCl irrigation + saline	56.2 ± 3.9	53.2 ± 6.7
NaOCl irrigation + k21	41.6 ± 9.7	48.3 ± 3.2
NaOCl irrigation + calcium hydroxide	49.3 ± 6.3	48.7 ± 5.7
NaOCl irrigation + chlorhexidine (CHX)	50.4 ± 3.6	49 ± 7.4
Saline	58.1 ± 2.9	64 ± 4.2
K21	51.6 ± 6.3	48.8 ± 1.2
Calcium hydroxide	55.0 ± 9	57.1 ± 8.9
Chlorhexidine (CHX)	56.9 ± 6	57.7 ± 1.8

**Fig. 4:**
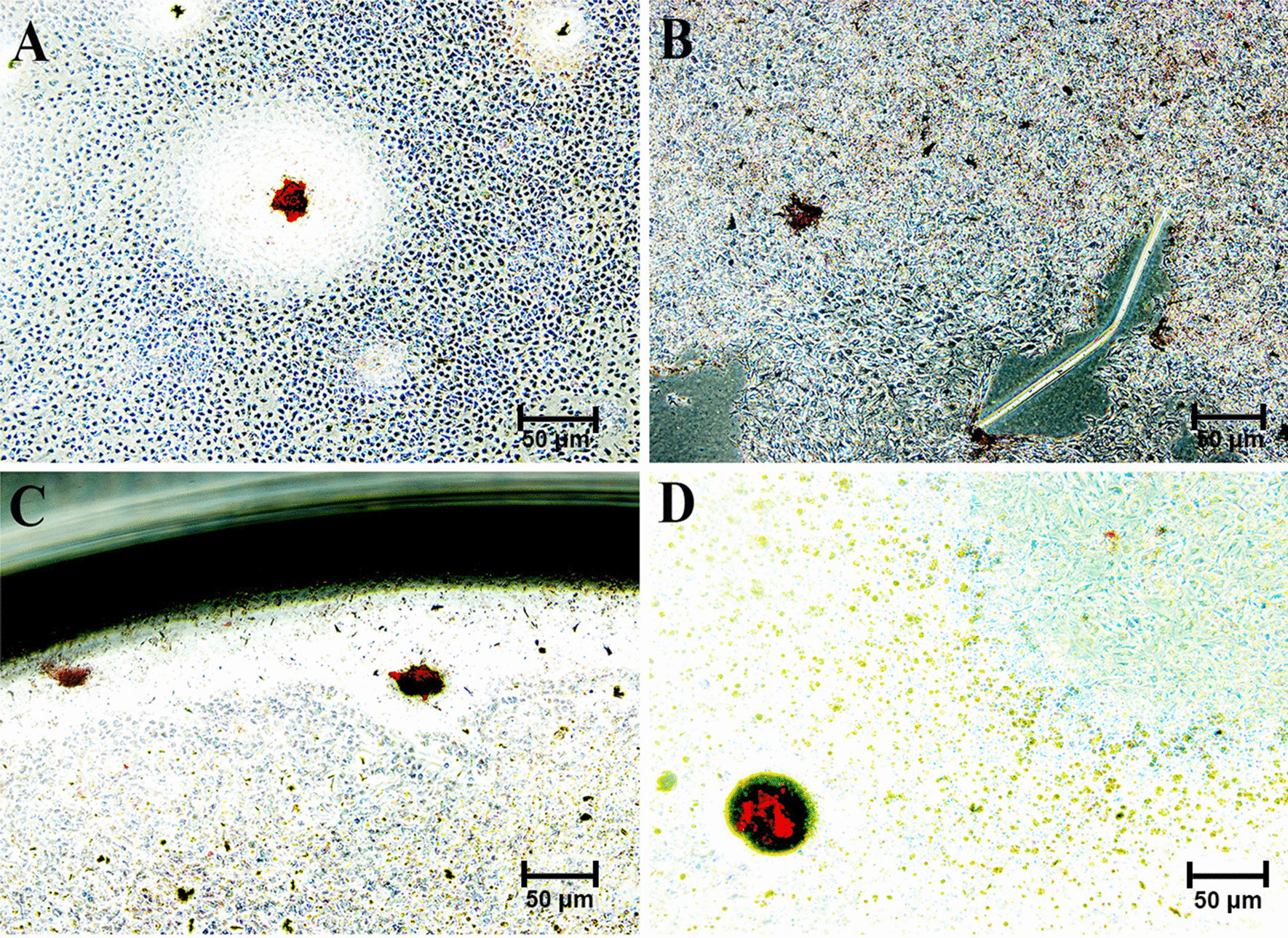
Alizarin red staining of human dental pulp stem cells (hDPSCs). Mineralized nodules as seen in light micrographs of human dental pulpal stem cell specimens treated with (**A**) no treatment as control; (**B**) 2%CHX; (**C**) 41% Ca(OH)2; (**D**) 2%k21. Lesser staining was observed after 3 days as compared to the 2%k21 specimens. Positive dark red staining shows presence of calcium deposits i.e. mineralization in the tested human dental pulpal stem cells.. Scale bar = 50 μm

**Fig. 5:**
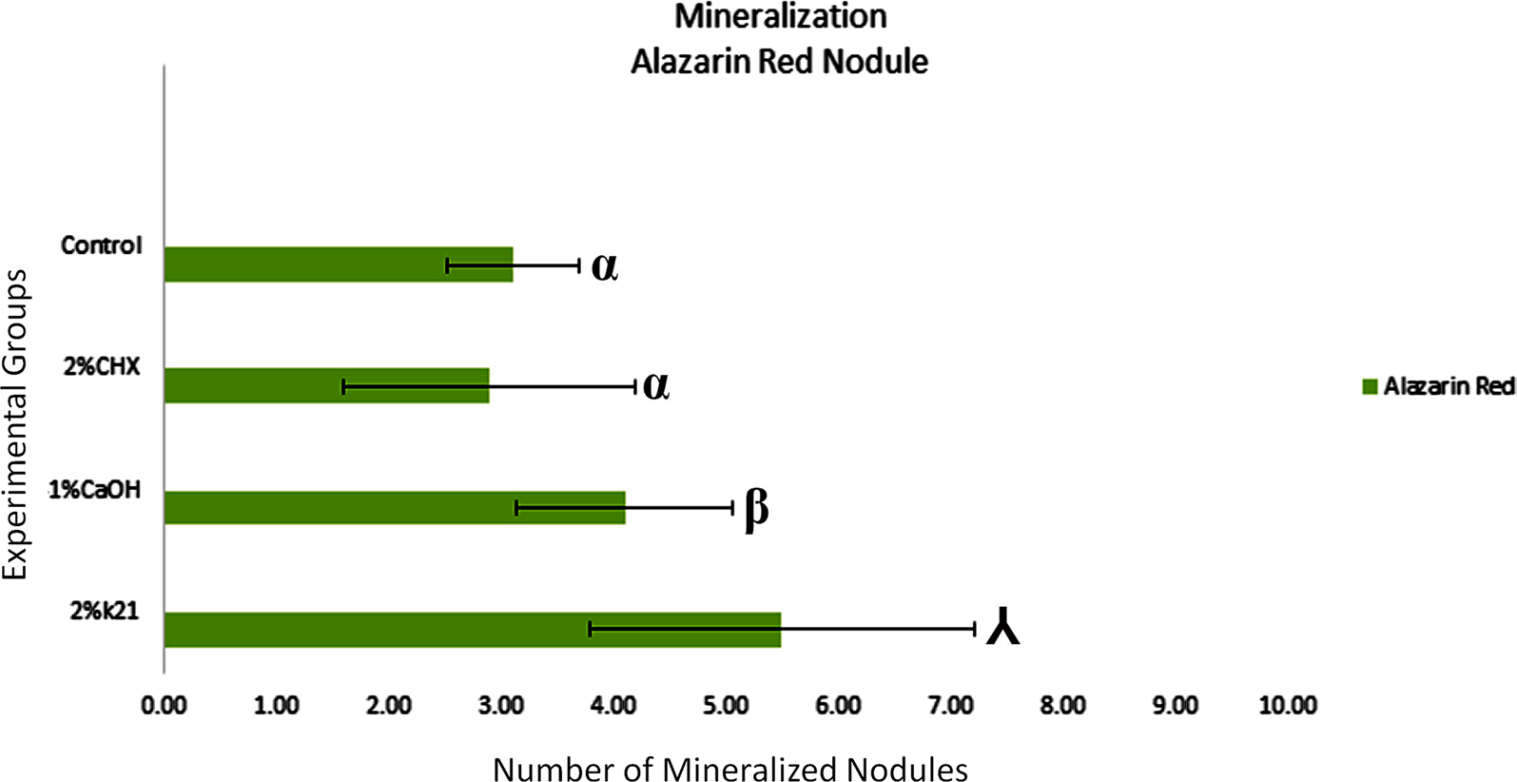
Mineralized nodule production by hDPSCs cells after application of intracanal medicaments on root dentine discs. Values are mean ±  standard deviation (n = 5). Groups identified by different symbols are not statistically different (*p* > 0.05)

Positive dark staining shows presence of calcium deposits i.e. mineralization (Fig. 4). Mineralized nodules are seen in light micrographs of human dental pulpal stem cell specimens treated with (A) no treatment as control groups (Fig. 4A); Alizarin red staining of the hDPSCs revealed the calcium deposition in all experimental groups cultured with osteogenic differentiation medium with significant difference seen amongst 2%k21 groups as seen after 3 days of culture. The factor disinfectant was considered significant (*p* < 0.05). No significant difference in mineralized nodule deposition was found between 2%CHX and control group (Fig. 5), with calcium deposits and staining positively with Alizarin Red in 2%k21. Small round Alizarin Red-positive nodules (Fig. 4D) formed more uniformly in 2%k21 specimens as compared to chlorhexidine (Fig. 4B) and calcium hydroxide (Fig. 4C) groups (*p* < 0.05). Variation between replicated cultures was minimal, with standard deviations rarely exceeding 6 of the mean in the mineralisation quantification of precipitated calcium (Fig. 5).

**Fig. 6:**
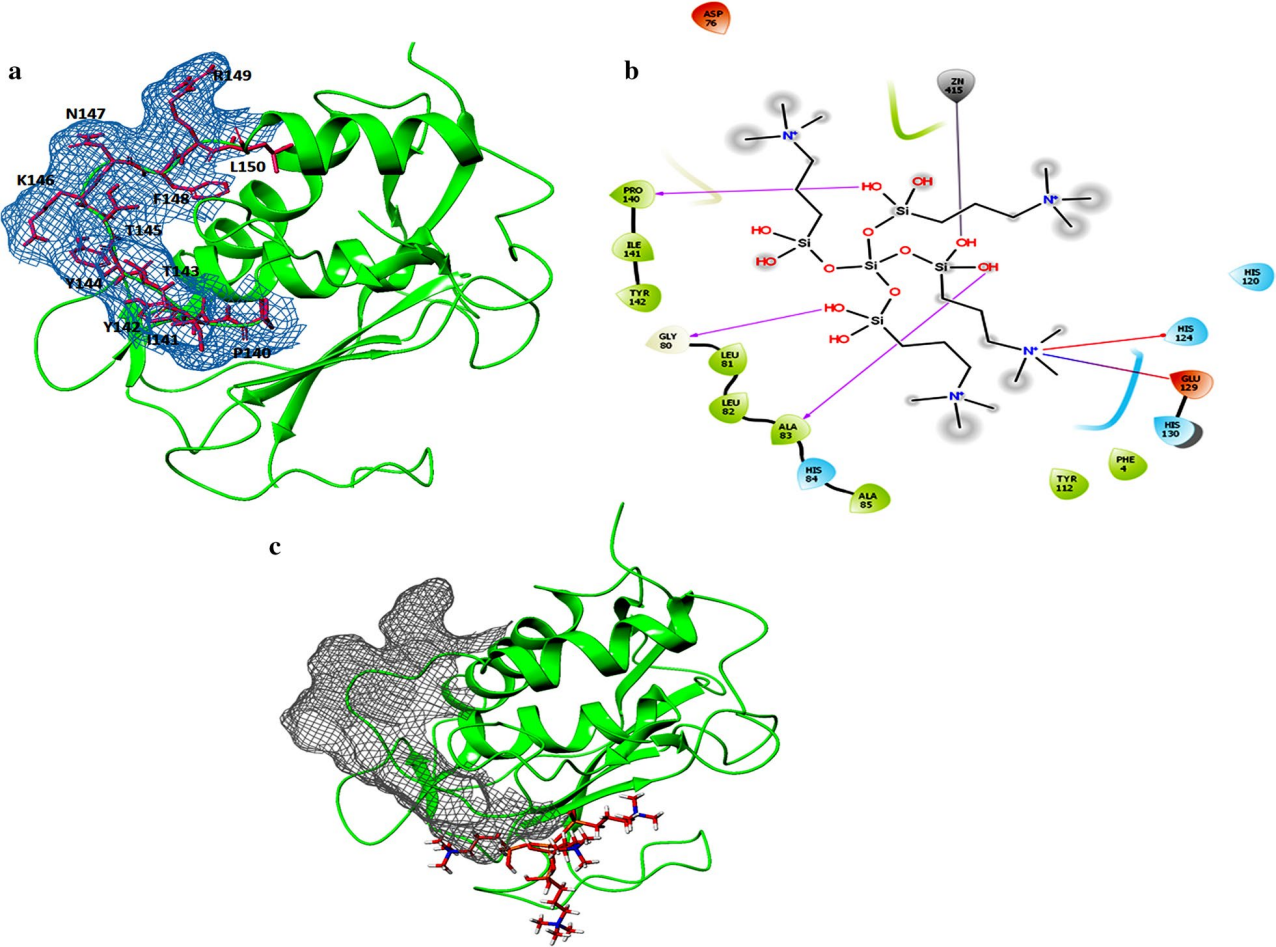
(**a**, **b**) The binding poses of quaternary ammonium compound in MMP-2 in both (**a**) 3D and (**b**) 2D. (**c**) MMP-2 is seen posed with K-21 chains up to quaternary nitrogen and associated with the silanol

A larger SiteScore (0.995352) was selected for MMP-2 binding pocket. The DScore of 1.019596 was selected for the attachment seen for the ligand binding accurately indicating the possible sites to be targets for k21 intervention (Fig. 6). The DScore determined massive propensity of k21 with the binding site with XP GScore of -2.593 with attachment at key amino acid levels. The results indicated that k21 may not sit in the S1 binding pocket of MMP-2. However, it still forms a bonding with zinc ion. The k21 compound shows a heavy ligand, making it far too difficult to dock the whole compound into the binding pockets of MMPs. Therefore, k21 ligand was split into different compounds.

## Discussion

*E. faecalis* has occasionally been found in cases of primary endodontic infections and frequently isolated root canal failed teeth [14, 15]. Moreover, Enterococci are known to survive in harsh environments which explains the persistence of root canal infections in presence of scarcely found nutrients and root canal medicaments [14, 15]. *E. faecalis* has been shown to invade dentinal tubules and colonize root canals by surviving without the support of other bacteria [14, 15, 39]. One of the most important consideration during root canal treatment is the eradication of microorganisms from the complex three-dimensional root canal system. This can mainly be achieved by efficient cleaning and shaping of the root canal system [40]. Due to complexity of the root canal morphology, hand and rotary instruments cannot clean areas such as the isthmuses, lateral canals, and dentinal tubules. The rotatory motion of the NiTi files enable a circular shape to the root canal space leaving behind unprepared lingual and buccal extensions favoring retention of bacterial remnants and necrotic tissue [41]. In such cases, effective irrigation, as well as the use of intracanal medicaments, is essential to reduce the microbial count in the root canal system [42–44].

Candida species contribute to an increase in the extracellular polymeric substances *(*EPS) of biofilms by changing the properties, virulence, and matrix structure within the biofilm [45]. Glycosyltransferase B can attach itself easily onto the surface of *Candida albicans* [46] producing a glucans-rich matrix. The infections produced by *C. albicans* are heterogeneous depending on the phenotype of a clinical isolate having profound outcomes [47]. Therefore, we sought to test whether these characteristics played a role in a dentally relevant scenario.

This study provides important information on the antimicrobial effects of k21 on both *E. faecalis* and *C. albicans* biofilms. Results from CLSM have shown that 2%k21 when used as an intracanal medicament, has a significant antimicrobial effect against both the organisms (Figs. 1, 2). The red fluorescence seen in CLSM images is due to the SYTO9 green fluorescent nucleic acid stain emission bands within the red wavelength [48]. The green fluorescence present within the control specimens indicated bacteria were mostly alive. There was a moderate decrease in the *C. albicans* biofilms (Fig. 2c) seen amongst chlorhexidine specimens as compared to the 41% calcium hydroxide (Fig. 2d) and 2%k21 (Fig. 2e) specimens. Hence the first null hypothesis must be accepted suggesting that 2%k21 has an antimicrobial effect on the *microbial* biofilm. There are reactive silanol groups in the new class of antimicrobials of organosilane quaternary ammonium salts. This is because of covalent bonding of Si–O linkages and hydrolysis of performing microbiocidal infections [49]. 3-(Trimethoxysilyl) propyldimethyloctadecyl ammonium chloride (SiQAC) penetrates the bacterial cell membranes via positively charged quaternary amine charged (N +) compounds leading to bacterial lysis due to their interaction with the negatively charged bacterial membranes [50]. Thus, the current study claimed that the microbial effectiveness of k21 at 2% as minimum concentration required to achieve the desired efficacy and hence could have overcome any shortcomings of existing disinfectants, as per our previous studies [24, 27]. Mono-species biofilms have been tested in our study, though studying more complex biofilms is the need of the hour. However, there is a difference of pathogenicity and survival in the in vivo multi-species plaque biofilms. Hence, it is difficult to corelate the in-vitro results to the in vivo situation and caution should be exercised in their interpretation.

Endodontic diseases are primarily due to biofilm infection, as bacteria can survive inside the root canal [51]. The incorporation of 2%k21 is itself an idea to prolong the antimicrobial effect without apparent decrease over a period. Along with the presence of tetraethoxysilane and functional organic groups, there were detectable dead bacterial cells seen in *E. faecalis* confocal specimens. This is due to immobilization of the SiQAC molecules forming a protective film resulting in circumventing and slowing down the growth of biofilms. Chlorhexidine forms precipitates and discoloration, as these precipitates are insoluble in water [52]. The time taken by microorganisms inside root canal system was more with chlorhexidine medicaments as compared to calcium hydroxide [53]. Calcium hydroxide has been shown to be ineffective at killing *E. faecalis* because of the resistance of *E. faecalis* against the medication and due to inadequate infiltration of medicament inside the dentinal tubules [54]. This is as a result of ions penetrating the cell membrane as well as the cytoplasm’s buffering capacity. However, calcium hydroxide specimens showed reduced number of bacteria after 7 days of growth. Until further studies are conducted, an intracanal dressing of 41% calcium hydroxide placed for 7 days appeared better to eradicate bacteria as compared to 2% chlorhexidine. In this study, we used a simple method of growing bacterial colonies on dentine specimens to evaluate the process of matrix degradation. The quality of the collagen structure was somewhat higher with no evidence of denaturation with specimens treated with 2%k21 (Fig. 2i). Collagen fibrils showed well-demarcated collagen cross banding pattern, when especially compared to 2% chlorhexidine (Fig. 2g) and control specimens (Fig. 2f). Furthermore, the isolated fibrils seen amongst chlorhexidine and calcium hydroxide treated specimens, revealed an irregular arrangement of substructures featuring an unevenness. The cell number was notably increased in control and 2% chlorhexidine specimens in contrast to the 2%k21 specimens, a fact that is coincident that there is incapability to form biofilm (Table 2). We interpreted this to mean that the collagenolytic activity that we measured due to bacterial organisms may have been reduced due to the disinfectants, especially in the 2%k21 group.

Components of exopolysaccharides are linked by glycosidic bonds of polysaccharides and carbohydrates [24, 26]. Raman effect produced by this bond was assigned to the shift region of 484 cm^−1^ to 498 cm^−1^ [24, 26]. This contains multidimensional information on special cellular components, even so indicating use of different medicament strategies marking a difference in intensities and deviations in structural elements with intensive signals at 484 cm^−1^ (Fig. 3a) in terms of arbitrary unit. Unlike proteins in EPS, exopolysaccharides are crucial for the maintenance of biofilm matrix, therefore the reduction in exopolysaccharides or lesser glycosidic bonds can suggest disruption of biofilm. In our research, the study of the ability and degree of disruption on the glycosidic band found in exopolysaccharides by intracanal medicaments is similar to the previous research regarding the ability of sodium hypochlorite in cleaving the same bond [26]. The reduction in the peak was seen highest in 2%k21 specimens as compared to chlorhexidine and calcium hydroxide medicament specimens (Fig. 3a). These Raman signatures by 3rd day was close to 0 arbitrary unit, hypothesizing a near complete eradication of biofilm upon the application. However, after re-introduction of nutrition into the dentine disc by returning dentin discs to orbital shaker with TSB medium for 4 days, bacteria biofilm started to form again. This was demonstrated by an increase in Raman intensity at 476cm^1^ region from 3rd to 7th day. Even though bacteria can grow after application of medicaments, the growth of bacteria biofilm in dentine disc treated by 2%k21 is the least, hypothesizing a stronger inhibition on growth of bacterial biofilm. Based on this compatibility, the least amount of intensity was found in 41% calcium hydroxide > followed by 2% chlorhexidine treated specimens. These changes are due to the Raman wave numbers and intensities which are produced due to the compositions and chemical environment, which in this case are the carbohydrate or biofilm signatures. This Raman mapping indicates a quantitative, amount and distribution of the change happening [55]. There was an overall net effect due to an increase in signal/noise ratio of these specific Raman peaks which were well within the estimated error of measurement. These changes were aligned to the structural perturbation within the biofilm. These changes are best understood because of the long hydrocarbon chains which enables a change in in hydrophobicity and surface energy. Within its cationic surfactant molecules, there is presence of lipophilic and hydrophilic groups [56] with antibacterial effect resulting due to electric charge inversion concentration. The electrostatic shield effect via Van der Waals forces of negatively charged bacterial cell are subdued decreasing the pathogenicity. Further detailing the mechanism, the hydrophobic interior of the cell membrane gets a charged interaction hydrocarbon tail of the cationic amphiphilic substance causing interpolation and eventual cell death [57]. Due to quaternization and surfactant properties, there is a protective coat formed on the dentin substrate, further subjugating the biofilm formation [58]. Raman intensity for 2% chlorhexidine on 3rd and 7th day at 483 cm^−1^ was higher in intensity than calcium hydroxide, suggestive of chlorhexidine being able to disrupt the bacteria biofilm at a degree lower than calcium hydroxide. The ability of chlorhexidine to disrupt but not destroy biofilm also coincided with researches mentioned above [59–61]. In addition to this, there was an interesting observation seen at the region of 588 cm^−1^. This region had been attributed to the beta-galactosidase membrane enzyme, most likely of the *E. faecalis biofilm*, a result of cell membrane lysis (Fig. 3b). The study provides a description of Raman scattering to detect galactosidase within bacterial cells in a nondestructive manner. The Raman analysis displayed improved sensitivity in an inherently weak process prone to interference from fluorescence. The presence of bacterial enzyme was detected due to a novel sharp vibrational Raman peak at 598 cm^−1^; the phospho-β-galactosidase found in bacterial cytoplasm [62]. For all experimental groups except control, Raman intensities at region 588 cm^−1^ had increased, suggestive that all medicaments are able to destroy the bacteria and causes leakage of beta-galactosidase. Moreover, 2%k21 (*p* < 0.05) showed no cytotoxic effects on cultured cells (Fig. 4) with no detrimental effect on mineralized nodule production. The normal deposition of mineralized nodules in 2%k21 (Fig. 4d) specimens indicated minimally affected cells, showing potential to repair the dentine pulp complex, which are essential tertiary dentin formation. Small round alizarin red-positive nodules were found to be significantly uniform in 2%k21 specimens as compared to chlorhexidine (Fig. 4b), calcium hydroxide (Fig. 4c) and control groups (Fig. 4a) (*p* < 0.05), rendering k21 as a safe medicament.

The microbial adhesion to solid surface is the first step for biofilm formation [63]. Studies showed that chemical agents have the ability to change the physicochemical properties of dentine which can influence the nature of adherence of microorganism to dentine [37, 64]. Hence investigating the influence of 2%K21 on the adherence of *E. faecalis* and *C. albicans* to root canal dentine would be of importance. From the adherence assay, the mean adherence values of *E. faecalis* and *C. albicans* to dentine treated with sodium hypochlorite and 2%k21 showed least adherence as compared to other medicaments, whereas saline showed highest adherence of *E. faecalis and C. albicans* to root canal dentine (Table 4). The quaternary ammonium ions disrupt the cytoplasmic membrane due to the presence of Si–O-Si or Si–O-C bonding mediating impeccable attachment and antibacterial potential [65]. Thus, the medicament can be rendered safe for use. The mean and standard deviations of log CFU counts for single -species biofilms following treatment with medicaments are shown in Table 1. The control saline specimens revealed the highest CFU value. The 2%CHX, 2%k21 and 41% calcium hydroxide produced similar log CFU counts, determining 2% k21 ability to perform appreciably along with other experimental disinfectants when compared to the control groups. Results from CFU have shown that 2%k21 has a significant antimicrobial effect against both bacterial biofilms, equivalent to the commonly used intracanal medicaments in Endodontics. In addition, the compound k21 resulted in a direct alignment of MMP-2 using molecular docking studies with collagen (PDB ID: 1Q7D) and binding pocket with appreciable SiteScore indicative of its multifaceted approach in maintaining not just the antimicrobial part, but also the matrix metallo proteinases (Fig. 6).

A dual species or multi complex biofilms may have an interaction between the colonies. Therefore, the results of *in-vitro* single species cannot be extrapolated to the in vivo situation and caution should be exercised in their interpretation. The quest for an ideal intracanal medicament for a sustained and efficient action against microbes has been ongoing for many years. Despite its effective results in the study, 2%k21 consideration as a constituent intracanal medicament requires more studies. From a practicing endodontist’s point of view, the current results strongly support the use and introduction of this medicament protocol as an alternative. The well-established use of calcium hydroxide maybe appropriate in most clinical endodontic cases, however, when confronted with a refractory periapical *E. faecalis* lesion or infection, a different intracanal medicament should be explored. The 2%k21 presented in this preliminary study has promising antimicrobial properties essential against *E. faecalis* and *C. albicans* infections, leading to discovery of new biologically active molecules. To standardize the duration between disinfection agents used in the study, we used calcium hydroxide for shorter duration. This will be considered as the potential limitation of the current study. However, the mode of action of k21 is very quick (20 s), therefore that was also a consideration while designing the experiment.

## Conclusion

Within the limitation of the current study, quaternary ammonium silane (2%k21) exhibited comparable antimicrobial activity when compared with chlorhexidine and calcium hydroxide as an intracanal medicament impregnated inside a root canal. Future clinical studies are required to confirm the results of the current in vitro study. Since the endodontic system is complex, penetrating dentinal tubules is difficult, and potential to exploit the k21 molecule as an intracanal medicament appears a feasible therapeutic approach against endodontic biofilm infection within the root canal system.

## Data Availability

The datasets generated and/or analysed during the current study are not publicly available due to the confidentiality of material development but are available from the corresponding author on reasonable request.

## Abbreviations

C.: Candida
CHX: Chlorhexidine
CLSM: Confocal Laser Scanning
E.: Enterococcus
IMU: International Medical University
MMPs: Matrix metalloproteinases
SDA: Sabouraud Dextrose Agar
SEM: Scanning Electron Microscopy
TEM: Transmission Electron Microscopy
TEOS: Tetraethoxysilane
